# Cognitive protection of incretin‐based therapies in patients with type 2 diabetes mellitus: A systematic review and meta‐analysis based on clinical studies

**DOI:** 10.1111/jdi.14015

**Published:** 2023-05-05

**Authors:** Sanbao Chai, Fengqi Liu, Shuqing Yu, Zhirong Yang, Feng Sun

**Affiliations:** ^1^ Department of Endocrinology and Metabolism Peking University International Hospital Beijing China; ^2^ Department of Epidemiology and Biostatistics, School of Public Health Peking University Health Science Center Beijing China; ^3^ Shenzhen Institute of Advanced Technology Chinese Academy of Sciences Shenzhen China

**Keywords:** Cognitive function, Incretin‐based therapy, Type 2 diabetes

## Abstract

**Aims/introduction:**

Cognitive dysfunction, including mild cognitive impairment and dementia, is increasingly recognized as an important complication of type 2 diabetes mellitus. The aims of the preset study was to investigate the cognitive protection of incretin‐based therapies, including glucagon‐like peptide‐1 receptor agonists and dipeptidyl peptidase‐4 inhibitors, in patients with type 2 diabetes mellitus.

**Materials and Methods:**

PubMed, EMBASE, Cochrane library, Web of Science and PsycINFO were searched from the inception through 17 January 2023 for randomized controlled trials and cohort studies on the association between incretin‐based therapies and cognitive function. A total of 15 studies were finally included in our systematic review, and eight of which were incorporated into our meta‐analysis.

**Results:**

Pooled results showed that the Mini‐Mental State Examination score in incretin‐based therapy groups was increased by 1.20 compared with the control group (weighted mean difference 1.20, 95% confidence interval 0.39–2.01). The results of eight studies assessed by the Newcastle Ottawa Quality Assessment Scale and the Cochrane Collaboration's tool, and the quality of the eight studies were at a relatively high level. Egger's regression did not show significant publication bias.

**Conclusions:**

Current evidence shows that incretin‐based therapies might be more effective, when compared with the other hypoglycemic drugs, for cognitive improvement in patients with type 2 diabetes mellitus.

## Graphical abstract

Cognitive dysfunction is recognized as an complication of type 2 diabetes mellitus. Incretin has emerged as a potential therapeutic agent for Alzheimer's disease. Incretin has a protective effect on cognitive function in type 2 diabetes mellitus patients.

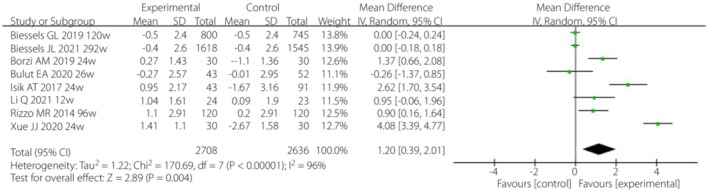

## INTRODUCTION

In the past decades, the prevalence of diabetes has risen rapidly and affected the quality of life of patients^1^. Cognitive dysfunction, including mild cognitive impairment and dementia, is increasingly recognized as an important complication of type 2 diabetes mellitus^2^. Approximately 10% of dementia patients have diabetes^3^. Compared with patients without diabetes, type 2 diabetes mellitus patients have a higher risk of cognitive impairment^4^, ^5^, especially when accompanied by renal^6^ and cardiovascular complications^7^. The English Longitudinal Study of Aging showed that cognitive decline was associated with prediabetes and diabetes, and the increment in hemoglobin A1c was significantly associated with an increased rate of decline in cognitive function^8^.

Incretin‐based therapies include glucagon‐like peptide‐1 receptor agonists (GLP‐1 RAs) and dipeptidyl peptidase‐4 (DPP‐4) inhibitors^9^, ^10^. Glucagon‐like peptide‐1 (GLP‐1) is an endogenous peptide hormone released by intestinal L‐cells in response to a meal. GLP‐1 stimulates insulin secretion from the pancreatic β‐cells under hyperglycemic conditions and reduces glucagon secretion from the α‐cells, recovering insulin sensitivity and enhancing glycemic homeostasis^11^. The effect of DPP‐4 inhibitor is indirectly mediated by GLP‐1. GLP‐1 is mainly metabolized through DPP‐4 enzyme degradation, which is expressed in many organs, such as the liver, pancreas, intestine and brain^12^, ^13^. Incretin‐based therapies are increasingly used in type 2 diabetes mellitus, and have also emerged as a potential therapeutic agent for Alzheimer's disease (AD)^14^, as well as vascular brain injury. Meta‐analysis based on randomized controlled trials shows that GLP‐1 RAs can significantly reduce the risk of stroke in patients with established atherosclerotic cardiovascular disease^15^, ^16^. Although some studies examined the association between incretin and cognitive function in type 2 diabetes mellitus, their findings were inconsistent^17^, ^18^, ^19^, ^20^, ^21^, ^22^, ^23^, ^24^, ^25^, ^26^, ^27^. Therefore, we carried out a systematic review and meta‐analysis to investigate the association between incretin‐based therapies and cognitive function in patients with type 2 diabetes mellitus.

## MATERIALS AND METHODS

The present study was carried out according to the Preferred Reporting Items for Systematic Reviews and Meta‐Analyses statement^28^. This study was registered on the International Prospective Register of Systematic Reviews (PROSPERO), and the registration number is CRD42020159750.

### Search strategy and literature search

We carried out literature searches from online databases of literature including: PubMed, EMBASE, Cochrane Library, Web of Science and PsycINFO. The search date ranges from the inception of each database through 17 January 2023. We used “Glucagon‐Like Peptide‐1 Receptor” and “Dipeptidyl‐Peptidase IV Inhibitors” as keywords or MeSH/EMTREE terms, accompanied with other relevant free words to search these databases; details of the search strategies are provided in Table S1.

### Literature screening and selection

Inclusion criteria included: (1) randomized controlled trials (RCTs) or cohort studies involving DPP‐4 inhibitors or GLP‐1 RAs compared with placebo or other antidiabetic agents (metformin, insulin, sulfonylureas, thiazolidinediones, alpha‐glucosidase inhibitors, sodium–glucose cotransporter 2 inhibitor) in patients with type 2 diabetes mellitus were included in our analysis (Table 1); and (2) eligible studies should also report any outcome of cognitive function, such as Trail Making Test‐A, Trail Making Test‐B, Montreal Cognitive Assessment, Digit Symbol Substitution Test, Cognitive Failures Questionnaire, Letter‐N‐Back and Cognitive Performance Scale. Exclusion criteria included: (1) studies not related to exposure or outcome; (2) studies for other drugs or other disease; (3) not original studies; (4) non‐English studies; and (5) studies for animals. Only studies using validated instruments to assess cognition (Mini‐Mental State Examination [MMSE]) were included in the present meta‐analysis. The eligibility of studies was assessed independently by three reviewers (SQY, SBC and FS), with any disagreement being resolved by consensus.

**Table 1:** Study characteristics of included studies

Study ID	Study design	Country	Incretin‐based therapies	Control	Sample size		Background medicine	Trial duration (week)	Outcome	Baseline information				
										Age (year)	MMSE		HbA1c (%)	Duration of type 2 diabetes mellitus (year)
					I	C					I	C		
Rizzo 2014^17^ ^†^	Retrospective cohort	Italy	Vildagliptin Sitagliptin Saxagliptin	Glimepiride Glyburide Glipizide	120	120	Metformin	96	MMSE TMT‐A TMT‐B	72.8	26.10	26.10	8.0	–
Isik 2017^18^ ^†^	Prospective cohort	Turkey	Sitagliptin	Insulin/metformin	101	104	–	24	MMSE	75.4	23.48	23.12	7.5	14.2
Biessels 2019^20^ ^†^	Randomized, double‐blind	Multiple countries	Linagliptin	Placebo	800	745	–	120	MMSE TMT‐A TMT‐B	67.8	28.3	28.2	7.8	15.1
Borzi 2019^21^ ^†^	Retrospective cohort	Italy	Vildagliptin	Metformin	30	30	Metformin	24	MMSE	76.6	21.00	20.77	7.6	16.1
Xue 2020^22^ ^†^	Randomized, clinical trial	China	Sitagliptin	Sulfonylurea	30	30	–	24	MMSE MoCA	68.0	25.42	25.37	8.7	8.6
Bulut 2020^23^ ^†^	Retrospective longitudinal	Turkey	Vildagliptin	Other hypoglycemic drugs	43	52	–	26	MMSE	74.3	21.81	26.04	7.4	17.4
Biessels 2021^26^ ^†^	Randomized double‐blind	Multiple countries	Linagliptin	Glimepiride	1,518	1,645	–	292	MMSE	64.4	28.5	28.5	7.1	7.6
Li 2021^27^ ^†^	Phase III trial	China	Liraglutide	Hypoglycemic drugs Insulin	24	23	–	12	Digit span test Total learning Long‐delay Free recall Recognition Animal naming test Clock Drawing test Trail Making test Minimum mental state examination Memory and executive Screening	57.3	41.0	40.4	9.1	9.3
Perna 2018^31^	Randomized clinical trial	Italy	Liraglutide Vildagliptin Sitagliptin Linagliptin	Canagliflozin Empagliflozin Dapagliflozin	18	21	Metformin	48	Verbal fluency test; Babcock Story Recall Test; Attentive Matrices Test	77.2	–	–	7.2	11.2
Nair 2019^19^	Cross‐sectional	India	DPP‐4 inhibitors	Sulfonylurea alpha‐glucosidase inhibitor thiazolidinedione	151	174	Metformin	–	MMSE	52.5	–	–	–	5.9
Cukierman‐Yaffe 2020^32^	Randomized, double‐blind	Multiple countries	Dulaglutide	Placebo	4,456	4,372	–	384	MoCA DSST	65.5^‡^	–	–	7.3	–
Jeong 2021^24^	Retrospective	Korea	DPP‐4 inhibitor	Other hypoglycemic drugs	52	56	–	256	MMSE	76.8	22.15	22.43	7.1	15.9
Secnik 2021^25^	Prospective cohort	Sweden	DPP‐4 inhibitor	Sulfonylurea Thiazolidinedione Insulin	103	389	–	48	MMSE	77.7	23^‡^	23^‡^	–	8.9
Eren‐Yazicioglu 2021^33^	Cross‐sectional	Turkey	Exenatide	Other hypoglycemic drugs	23	20	–	12	CFQ LNB	53.5	–	–	–	10.9
Zullo 2022^34^	Retrospective cohort	USA	DPP4 inhibitor	Sulfonylureas	892	892	–	24	CPS	80.5	–	–	7.8	–

C, control group; CFQ, Cognitive Failures Questionnaire; CPS, Cognitive Performance Scale; DSST, Digit Symbol Substitution Test; I, international group; LNB, Letter‐N‐Back; MMSE, Mini‐Mental State Examination; MoCA, Montreal Cognitive Assessment; TMT‐A, Trail Making Test‐A; TMT‐B, Trail Making Test‐B.

^†^ Trial included in meta‐analysis.

^‡^ Median.

### Data extraction

Data from studies selected for full‐text review were extracted in duplicate for quality assessment and data analysis. Data were extracted by using Excel (Microsoft, Redmond, WA, USA), including study information (author, publication year, sample size, trial duration, types of intervention and control), population characteristics (background therapy, diabetes duration, age, baseline level of hemoglobin A1c), reported outcomes of cognitive function and information of methodology.

### Quality and risk of bias assessment

We assessed the quality and risk of bias of these included studies by using appropriate tools based on the type of studies. The Newcastle Ottawa Quality Assessment Scale and the Cochrane Collaboration's tool were used for evaluating cohort studies^29^ and RCTs^30^, respectively. The Grading of Recommendations Assessment, Development and Evaluation (GRADE) framework was also used to assess the quality of evidence for cognitive function, which assessed the within‐study limitations, imprecision, heterogeneity, indirectness and publication bias for the MMSE outcome. The whole assessment process was completed independently by FQL and SBC in parallel, and FS confirmed the final extraction when there was any inconsistency.

### Statistical analysis

After screening and data extraction, eligible data were pooled through meta‐analysis carried out using Review Manager (5.3 version; Cochrane Collaboration, London, UK). The results of the comparison of cognitive function between the intervention and control group are shown as the mean difference (MD) of the MMSE score and its 95% confidence interval (95% CI). The heterogeneity of these studies was measured by the *I* ^2^‐statistic. Egger's regression was carried out to test publication bias because of small‐study effects. We carried out a sensitivity analysis including RCTs and cohort studies. We did not carry out further subgroup analysis or meta‐regression to explore potential sources of heterogeneity given the insufficient number of included studies.

## RESULTS

### Literature search

**Figure 1:**
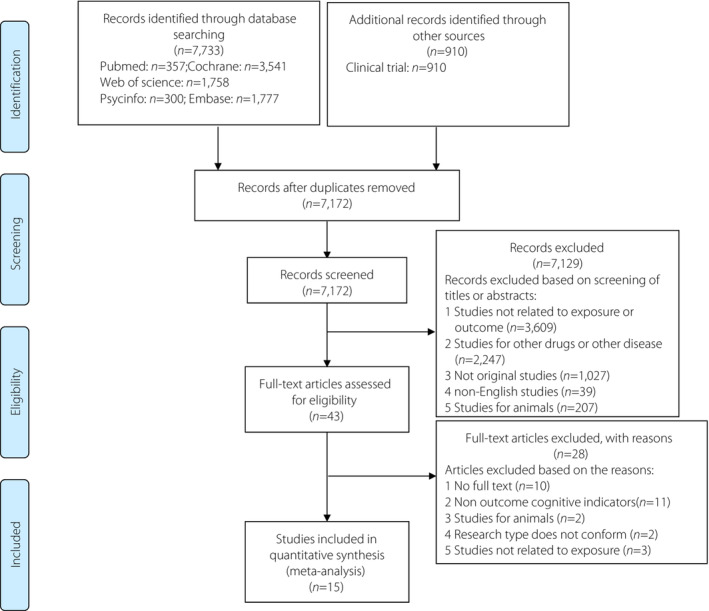
Flow chart of studies considered for inclusion.

Our search strategy resulted in the identification of 7,172 articles (Figure 1). After the extensive review of the titles and abstracts of these articles, 43 articles were identified for full‐text review. A total of 15 studies were finally included in our systematic review, and eight of them were incorporated into our meta‐analysis.

### Study characteristics

A total of 15 studies evaluated the association of incretin‐based therapies on cognitive function in patients with type 2 diabetes mellitus^17^, ^18^, ^19^, ^20^, ^21^, ^22^, ^23^, ^24^, ^25^, ^26^, ^27^, ^31^, ^32^, ^33^, ^34^, with eight included in our meta‐analysis^17^, ^18^, ^20^, ^21^, ^22^, ^23^, ^26^, ^27^. Of the eight eligible studies, four were RCTs and four were cohort studies. These studies were published between 2014 and 2021, and the total number of participants was 5,415 (3,259 men [60%]). Duration of follow up ranged from 12 to 292 weeks. There was no background drug in six studies, and metformin was the background drug in two studies. Seven studies were not included in our meta‐analysis. Of these seven studies, three of these studies did not have specific values for MMSE^19^, ^24^, ^25^, and four of these studies used other cognitive evaluation tools^31^, ^32^, ^33^, ^34^, respectively. Detailed information of 14 studies is showed in Table 1.

### Incretin‐based therapies and cognitive function

**Figure 2:**
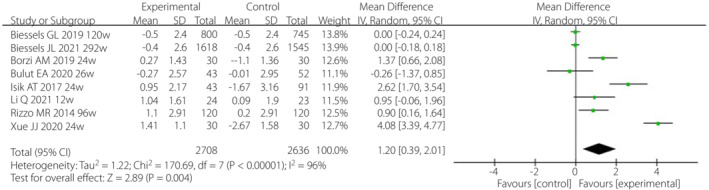
Association of incretin‐based therapies and cognitive function in patients with type 2 diabetes mellitus. CI, confidence interval; SD, standard deviation.

Pooled results showed that the MMSE score in incretin‐based therapy groups was increased by 1.20 compared with the control group (weighted MD [WMD] 1.20, 95% CI 0.39–2.01; Figure 2). Sensitivity analysis was carried out for four RCTs and four cohort studies, and the results were WMD 1.21 (95% CI 0.07–2.35) and WMD 1.19 (95% CI 0.19–2.19), respectively (Figure S1). The results of the sensitivity analysis were extremely close to the results of the main analysis.

One of the three RCTs showed positive results (WMD 4.08, 95% CI 3.39–4.77)^22^ and the other two showed negative results^20^, ^26^. Three of the four cohort studies showed positive results, and one showed a negative result. Of the four studies evaluated by other cognitive tools, two studies suggested that the use of incretin drugs could improve the cognition of patients with type 2 diabetes mellitus, and two studies showed no improvement in cognition performance or cognition function. The remaining three studies without MMSE baseline or specific values suggested that DPP‐4 inhibitors can significantly improve cognitive function in patients with type 2 diabetes mellitus compared with other oral hypoglycemic agents.

### Subgroup analysis of sulfonylureas and other oral hypoglycemic drugs

Meta‐analysis was carried out for the study of sulfonylureas as the control group and the study of non‐sulfonylureas as the control group, and the results were WMD 1.65, 95% CI −0.80 to 4.09 and WMD 0.93, 95% CI −0.09 to 1.94, respectively. The stratification result is consistent with the general conclusion. See Figure S2.

### Quality and risk of bias assessment results

The results of eight studies assessed by the Newcastle Ottawa Quality Assessment Scale and the Cochrane Collaboration's tool are shown in Tables S2 and S3. The quality of the eight studies included in the meta‐analysis were at a relatively high level. Egger's regression did not show significant publication bias. Publication bias refers to the research results with statistical significance being more likely to be reported and published than the results without statistical significance and that are invalid. According to GRADE, the quality of evidence was rated as high for the cognitive function outcome in the comparison between incretin‐based therapies and control groups (Table 2).

**Table 2:** Evaluation of the quality of evidence using the Grading of Recommendations Assessment, Development and Evaluation framework for the Mini‐Mental State Examination

Incretin‐based therapies compare to control for type 2 diabetes mellitus						
Patient or population: patients with type 2 diabetes mellitus						
Settings: multiple countries						
Intervention: incretin‐based therapies						
Comparison: control						
**Outcomes**	**Illustrative comparative risks** **†** **(95% CI)**		**Relative effect (95% CI)**	**No. Participants (studies)**	**Quality of the evidence (GRADE)**	**Comments**
	**Assumed risk**	**Corresponding risk**				
	**Control**	**Incretin‐based therapies**				
Cognitive function MMSE scores	The mean cognitive function in the control groups was 0 point	The mean cognitive function in the intervention groups was 1.20 higher (0.39–2.01 higher)	0.39, 2.01	5,415 (8 studies)	⊕ ⊕ ⊝ Moderate	

Mini‐Mental State Examination (GRADE) Working Group grades of evidence: High quality: Further research is very unlikely to change our confidence in the estimate of effect. Moderate quality: Further research is likely to have an important impact on our confidence in the estimate of effect and may change the estimate. Low quality: Further research is very likely to have an important impact on our confidence in the estimate of effect and is likely to change the estimate. Very low quality: We are very uncertain about the estimate.

CI, confidence interval; GRADE, Grading of Recommendations Assessment, Development and Evaluation; MMSE, Mini‐Mental State Examination.

^†^ The basis for the assumed risk (e.g. the median control group risk across studies) is provided in footnotes. The corresponding risk (and its 95% confidence interval) is based on the assumed risk in the comparison group and the relative effect of the intervention (and its 95% confidence interval).

## DISCUSSION

The present meta‐analysis showed that incretin‐based therapies improve cognitive function (evaluated using MMSE) in patients with type 2 diabetes mellitus. In our review, the significant association between incretin‐based therapies and cognitive function was observed in RCTs and observational studies. The overall quality of the studies included in the meta‐analysis is high.

The MMSE is a cognitive function assessment with 30 questions, involving six aspects: orientation, registration, attention and calculation, memory, language and visual spatial ability^35^. The MMSE is the most famous and commonly used screening tool for cognitive assessment in clinical and scientific research^36^, ^37^. The score is calculated based on the scores of correct items, and the score of missing items is 0^38^. Through verification, it is suggested that there is a high correlation between MMSE scores and other cognitive indicators and the scores of activities of daily living. It suggests that there is a high correlation between MMSE and other cognitive indicators, as well as activities of daily living^39^.

In addition to the common microvascular and macrovascular complications, cognitive decline is considered as an emerging consequence of type 2 diabetes mellitus^40^. Diabetes is a major risk factor for AD, vascular dementia and Parkinson's disease (PD). These neurodegenerative diseases are closely related to impaired glucose metabolism in diabetes. Among the eight trials included in the present meta‐analysis, the results of six trials^17^, ^18^, ^21^, ^22^, ^23^, ^27^ suggested that incretin drugs have the effect of improving the cognition of diabetes patients, and the results of two trials^20^, ^26^ were negative. Three trials suggested that the blood glucose of the incretin group was significantly lower than that of the control group^17^, ^20^, ^23^, and four trials suggested that there was no significant difference between the two groups^18^, ^21^, ^26^, ^27^. In terms of hypoglycemic events, all results suggested that the risk of hypoglycemia in the incretin group was significantly reduced. Therefore, controlling blood glucose to reach the standard and avoiding hypoglycemic events might improve the cognitive function of diabetes patients.

Insulin resistance and brain insulin signal transduction defects can accelerate the onset of neurodegenerative diseases by reducing brain metabolism. Although an association between diabetes and cognitive dysfunction has been observed, the effect of hypoglycemic drugs on cognitive dysfunction is still unclear. GLP‐1, as a physiological regulator of the central nervous system, enhances learning and memory functions by restoring insulin signal transduction^41^. Increasing evidence shows that GLP‐1 analogs have many extrapancreatic effects. GLP‐1 can affect cellular signal transduction through the blood–brain barrier, such as neuroinflammation and mitochondrial function in the central nervous system^42^. An open trial of exenatide in the treatment of moderate PD showed continuous improvement in motor and cognitive functions of patients treated with exenatide^43^. A double‐blind trial using exenatide weekly preparation showed that exenatide had a positive effect on off‐medication motor scores in patients with moderate PD^14^. Dumbrill and Moulton^44^ carried out a systematic review examining the effects of incretin‐based therapies on neurocognitive outcomes in human studies. The results showed that there was evidence to support that DPP‐4 inhibitor can improve cognitive function of patients, whereas GLP‐1 can improve cerebral glucose metabolism^44^. In a randomized, placebo‐controlled, double‐blind study, liraglutide improved glucose metabolism and cognitive function in AD patients^45^. The results of clinical research on incretin‐based therapies are consistent with those of preclinical research. Xue *et al*.^22^ found that treatment with DPP‐4 inhibitor for 6 months significantly improved the cognitive ability and increased the Aβ1‐42/Aβ1‐40 value in elderly patients with type 2 diabetes mellitus combined with post‐stroke mild cognitive impairment. These results suggested that DPP‐4 inhibitor could improve the cognitive function in patients with type 2 diabetes mellitus, which might be associated with the improvement of Aβ gathering.

In animal models, incretin‐based therapies can improve the pathological changes of AD. Some studies have shown that GLP‐1 can reduce oxidative stress and inflammatory reaction of neurons. The study of a PD mouse model found that GLP‐1 can maintain synaptic plasticity, thereby improving the motor and non‐motor deficits^42^, ^46^. In the diabetes rat model of cerebral ischemia/reperfusion injury, recombinant GLP‐1 improves neural injury by inhibiting oxidative stress response^47^. Using streptozotocin to prepare animal models of diabetes and cognitive decline, it was found that liraglutide could improve the hippocampal neurodegeneration of animals^48^. In mice models of AD, it was found that the treatment of sitagliptin and linagliptin could increase the level of GLP‐1 in the brain, thereby reducing memory impairment, tau phosphorylation and neuroinflammatory reaction^49^, ^50^. A study on AD transgenic mice found that intraperitoneal injection of exenatide can reduce the pathological changes in the hippocampus of mice, thereby improving cognitive function^51^. In the rat model of PD, vildagliptin plays a variety of neuroprotective effects, thereby improving the cognitive function of rats^52^.

Compared with Luan *et al*.^53^ and Jin *et al*.^54^, the novelty of this meta‐analysis includes the following three aspects. First, our research includes GLP‐1 RA_S_ and DPP‐4 inhibitors, and the drug is more comprehensive. Second, 15 studies were included in this meta‐analysis, including four RCTs. The quantity and quality of the studies were better than those of the aforementioned two studies. Third, in addition to the MMSE and Montreal Cognitive Assessment, the indicators of cognitive evaluation include the Trail Making Test, Verbal Fluency Test, Babcock Story Recall Test, Babcock Story Recall Test and Attention Matrices Test. The indicators of cognitive function evaluation are more comprehensive. Therefore, the results of the present study on the cognitive function of incretin in diabetes patients are more objective than those of the aforementioned two studies, which provides a basis for more clinical studies on the cognitive function of such drugs and diabetes patients.

There were several limitations to the present review. First, the overall quality of the evidence was assessed as moderate in the GRADE framework, so current evidence suggests an association of incretin‐based therapies with cognitive function, but cannot confirm the causal effects of the therapies on cognitive function. Second, there was substantial heterogeneity in the effect estimates. Different study types might be one of the possible explanations. Other possible reasons for heterogeneity could include the differences in study population, treatment duration, length of follow up and methodological quality. Due to the limited number of eligible studies, however, we could not carry out further subgroup analysis or meta‐regression to explore these potential sources of heterogeneity. Third, our study was limited to studies published in English only. Fourth, due to the small number of studies finally included, there will inevitably be some publication bias.

The result of the present meta‐analysis showed that incretin‐based therapies are associated with the reduced risk of cognitive impairment in patients with type 2 diabetes mellitus. Due to the limited number of RCTs included in this study and the unstable results, it is necessary to refer to the results of this study with caution to guide clinical practice. However, the conclusion of RCTs in this study is basically consistent with that of prospective cohort studies, which also increases the confidence of using this evidence. Further high‐quality studies are required to confirm the present findings.

## DISCLOSURE

The authors declare no conflict of interest.

Approval of the research protocol: N/A.

Informed consent: N/A.

Registry and the registration no. of the study/trial: N/A.

Animal studies: N/A.

## Supporting information


**Figure S1** | Sensitivity analysis of randomized controlled trials and cohort studies.

**Figure S2** | Subgroup analysis of sulfonylureas and other oral hypoglycemic drugs as the control group.

**Table S1** | Search strategies.

**Table S2** | Cohort studies quality and risk of bias assessment (Newcastle Ottawa Quality Assessment Scale).

**Table S3** | Randomized controlled trials quality and risk of bias assessment (the Cochrane Collaboration's tool).
