# Supplementary material for: Cognitive protection of incretin‐based therapies in patients with type 2 diabetes mellitus: A systematic review and meta‐analysis based on clinical studies
Source: J Diabetes Investig. 2023 May 5;14(7):864–73. doi: 10.1111/jdi.14015 (PMC10286783; doi:10.1111/jdi.14015)
Supplement: Supplementary file 1 — Figure S1 | Sensitivity analysis of randomized controlled trials and cohort studies. [file JDI-14-864-s003.pptx]

## Slide 1
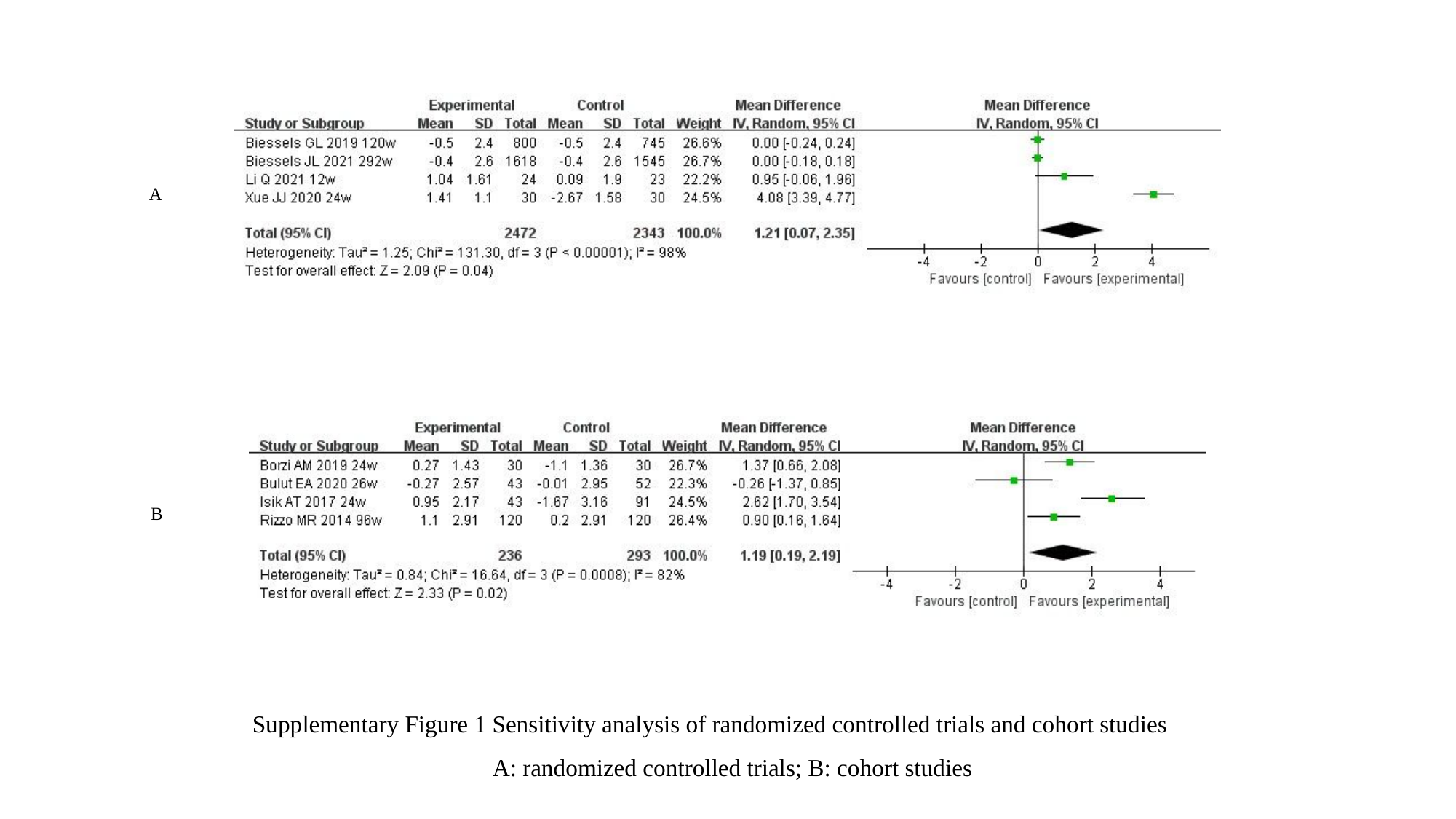

A
 B
Supplementary Figure 1 Sensitivity analysis of randomized controlled trials and cohort studies
 A: randomized controlled trials; B: cohort studies
