# Supplementary material for: Cognitive protection of incretin‐based therapies in patients with type 2 diabetes mellitus: A systematic review and meta‐analysis based on clinical studies
Source: J Diabetes Investig. 2023 May 5;14(7):864–73. doi: 10.1111/jdi.14015 (PMC10286783; doi:10.1111/jdi.14015)
Supplement: Supplementary file 2 — Figure S2 | Subgroup analysis of sulfonylureas and other oral hypoglycemic drugs as the control group. [file JDI-14-864-s004.pptx]

## Slide 1
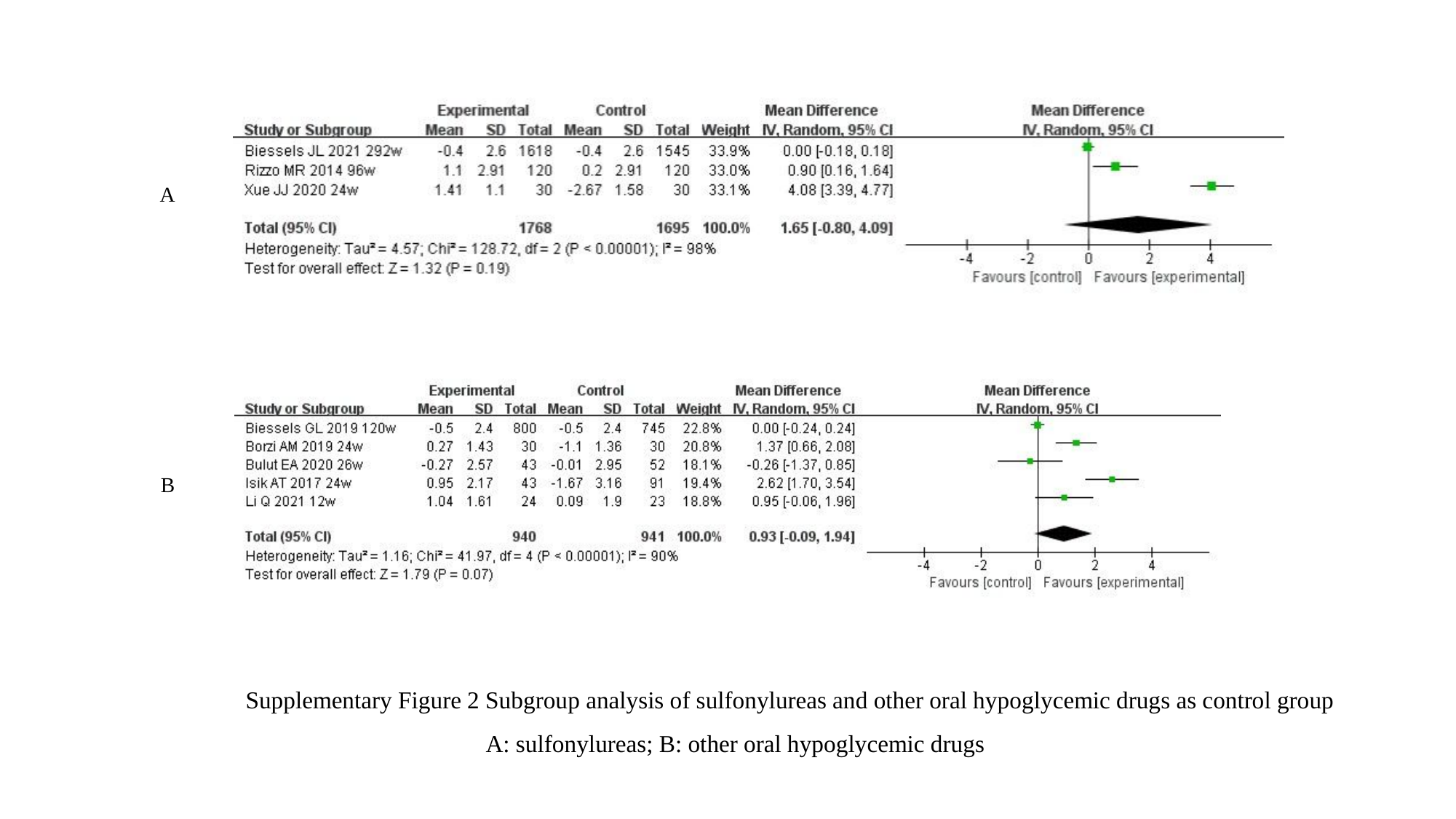

A
B
Supplementary Figure 2 Subgroup analysis of sulfonylureas and other oral hypoglycemic drugs as control group
 A: sulfonylureas; B: other oral hypoglycemic drugs
