# Supplementary material for: Cognitive protection of incretin‐based therapies in patients with type 2 diabetes mellitus: A systematic review and meta‐analysis based on clinical studies
Source: J Diabetes Investig. 2023 May 5;14(7):864–73. doi: 10.1111/jdi.14015 (PMC10286783; doi:10.1111/jdi.14015)
Supplement: Supplementary file 3 — Table S1 | Search strategies. [file JDI-14-864-s001.docx]

Supplementary Table 1 Search strategies

**Pubmed**

| **No.** | **Search strategies** |
| --- | --- |
| #1 | *Glucagon-Like Peptide-1 Receptor/ or glucagon-like peptide-1 agonists.mp. or *Glucagon-Like Peptide 1/ |
| #2 | glucagon like peptide*.ti,ab. |
| #3 | Receptors, Glucagon/ag [Agonists] |
| #4 | exenatide.af. |
| #5 | liraglutide.af. |
| #6 | albiglutide.af. |
| #7 | taspoglutide.af. |
| #8 | lixisenatide.af. |
| #9 | dulaglutide.af. |
| #10 | semaglutide.af. |
| #11 | Byetta.af. |
| #12 | Bydureon.af. |
| #13 | Victoza.af. |
| #14 | Lyxumia.af. |
| #15 | Adlyxin.af. |
| #16 | Tanzeum.af. |
| #17 | Eperzan.af. |
| #18 | Trulicity.af. |
| #19 | ZP10A peptide*.af. |
| #20 | "AVE 0010".af. |
| #21 | Ozempic.af. |
| #22 | GLP 1 Receptor Agonist*.af. |
| #23 | GLP 1 RA*.af. |
| #24 | GLP 1RA*.af. |
| #25 | 1 or 2 or 3 or 4 or 5 or 6 or 7 or 8 or 9 or 10 or 11 or 12 or 13 or 14 or 15 or 16 or 17 or 18 or 19 or 20 or 21 or 22 or 23 or 24 |
| #26 | Dipeptidyl Peptidase 4 inhibitor.mp. or exp *Dipeptidyl-Peptidase IV Inhibitors/ |
| #27 | dipeptidyl peptidase IV inhibit*.af. |
| #28 | Dipeptidyl Peptidase 4 Inhibit*.af. |
| #29 | DPP 4 inhibit*.af. |
| #30 | DPP4 inhibit*.af. |
| #31 | DPP4i.af. |
| #32 | DPP IV inhibit*.af. |
| #33 | DPPIV inhibit*.af. |
| #34 | sitagliptin.af. |
| #35 | Januvia.af. |
| #36 | Janumet.af. |
| #37 | Juvisync.af. |
| #38 | vildagliptin.af. |
| #39 | Galvus.af. |
| #40 | Eucreas.af. |
| #41 | "Galvus Met".af. |
| #42 | saxagliptin.af. |
| #43 | Onglyza.af. |
| #44 | "Kombiglyze XR".af. |
| #45 | Qtern.af. |
| #46 | alogliptin.af. |
| #47 | Nesina.af. |
| #48 | Oseni.af. |
| #49 | Kazano.af. |
| #50 | Vipidia.af. |
| #51 | Vipdoment.af. |
| #52 | linagliptin.af. |
| #53 | Trajenta.af. |
| #54 | gemigliptin.af. |
| #55 | Gemiglo.af. |
| #56 | anagliptin.af. |
| #57 | Beskoa.af. |
| #58 | teneligliptin.af. |
| #59 | Tenelia.af. |
| #60 | Diabegliptin.af. |
| #61 | Elant.af. |
| #62 | Glucal.af. |
| #63 | Teneglucon.af. |
| #64 | Veriglip.af. |
| #65 | Glipten.af. |
| #66 | Trelagliptin.af. |
| #67 | "PF 734200".af. |
| #68 | retagliptin.af. |
| #69 | Melogliptin.af. |
| #70 | evogliptin.af. |
| #71 | Suganon.af. |
| #72 | Carmegliptin.af. |
| #73 | "LC15 0444".af. |
| #74 | DA-1229.af. |
| #75 | omarigliptin.af. |
| #76 | gliptin*.af. |
| #77 | dutogliptin.af. |
| #78 | or/26-77 |
| #79 | 25 or 78 |
| #80 | (clinical trial or controlled clinical trial or randomized controlled trial).pt. |
| #81 | clinical trials.mp. or exp *Clinical Trial/ |
| #82 | clinical trials as topic/ or controlled clinical trials as topic/ or randomized controlled trials as topic/ |
| #83 | random*.ti,ab. |
| #84 | clinical trial*.ti,ab. |
| #85 | controlled trial*.ti,ab. |
| #86 | case-control studies/ |
| #87 | retrospective studies/ |
| #88 | cohort studies/ |
| #89 | longitudinal studies/ |
| #90 | follow-up studies/ |
| #91 | prospective studies/ |
| #92 | cohort.ti,ab. |
| #93 | longitudinal.ti,ab. |
| #94 | follow up.ti,ab. |
| #95 | followup.ti,ab. |
| #96 | prospective*.ti,ab. |
| #97 | retrospective*.ti,ab. |
| #98 | nonrandom*.ti,ab. |
| #99 | comparison group*.ti,ab. |
| #100 | control group*.ti,ab. |
| #101 | database*.ti,ab. |
| #102 | population*.ti,ab. |
| #103 | registries/ |
| #104 | registries.ti,ab. |
| #105 | trial$1 register.ti. |
| #106 | trial$1 registers.ti. |
| #107 | or/80-106 |
| #108 | 25 and 107 |
| #109 | 78 and 107 |
| #110 | 79 and 107 |
| #111 | limit 108 to humans |
| #112 | limit 108 to animals |
| #113 | 112 not 111 |
| #114 | 108 not 113 |
| #115 | limit 109 to humans |
| #116 | limit 109 to animals |
| #117 | 116 not 115 |
| #118 | 109 not 117 |
| #119 | limit 110 to humans |
| #120 | limit 110 to animals |
| #121 | 120 not 119 |
| #122 | 110 not 121 |
| #123 | 114 or 118 |
| #124 | meta analysis.pt. |
| #125 | Meta-Analysis as Topic/ |
| #126 | meta analy*.ti. |
| #127 | metaanaly*.ti. |
| #128 | 124 or 125 or 126 or 127 |
| #129 | 25 and 128 |
| #130 | 78 and 128 |
| #131 | 79 and 128 |
| #132 | limit 129 to humans |
| #133 | limit 129 to animals |
| #134 | 133 not 132 |
| #135 | 129 not 134 |
| #136 | limit 130 to humans |
| #137 | limit 130 to animals |
| #138 | 137 not 136 |
| #139 | 130 not 138 |
| #140 | limit 131 to humans |
| #141 | limit 131 to animals |
| #142 | 141 not 140 |
| #143 | 131 not 142 |
| #144 | 114 not 135 |
| #145 | 118 not 139 |
| #146 | 122 not 143 |
| #147 | 144 or 145 |
| #148 | remove duplicates from 144 |
| #149 | remove duplicates from 145 |
| #150 | Alzheimer Disease/ or 'cerebral glucose metabolism'.mp. |
| #151 | Cognitive Dysfunction/ or Cognition Disorders/ or Dementia/ or 'cognitive impairment'.mp. or Brain/ or Alzheimer Disease/ |
| #152 | dementia.mp. or Dementia/ or "Mental Status and Dementia Tests"/ or Frontotemporal Dementia/ or Dementia, Vascular/ or Dementia, Multi-Infarct/ |
| #153 | Dementia/ or Dementia, Vascular/ or Cognition Disorders/ or 'vascular dementia'.mp. or Alzheimer Disease/ |
| #154 | 150 or 151 or 152 or 153 |
| #155 | 108 and 154 |
| #156 | 109 and 154 |
| #157 | 110 and 154 |

**Cochrane**

| **No.** | **Search strategies** |
| --- | --- |
| #1 | MeSH descriptor: [Glucagon-Like Peptide 1] explode all trees and with qualifier(s): [administration & dosage - AD, adverse effects - AE, antagonists & inhibitors - AI, drug effects - DE, metabolism - ME, pharmacokinetics - PK, pharmacology - PD, therapeutic use - TU, toxicity - TO, agonists - AG] |
| #2 | MeSH descriptor: [Exenatide] explode all trees and with qualifier(s): [administration & dosage - AD, adverse effects - AE, metabolism - ME, pharmacokinetics - PK, pharmacology - PD, therapeutic use - TU, toxicity - TO] |
| #3 | MeSH descriptor: [Liraglutide] explode all trees and with qualifier(s): [administration & dosage - AD, adverse effects - AE, metabolism - ME, pharmacokinetics - PK, pharmacology - PD, therapeutic use - TU, toxicity - TO] |
| #4 | albiglutide |
| #5 | taspoglutide |
| #6 | lixisenatide |
| #7 | dulaglutide |
| #8 | semaglutide |
| #9 | #1 or #2 or #3 or #4 or #5 or #6 or #7 or #8 |
| #10 | MeSH descriptor: [Dipeptidyl-Peptidase IV Inhibitors] explode all trees and with qualifier(s): [administration & dosage - AD, adverse effects - AE, metabolism - ME, pharmacokinetics - PK, pharmacology - PD, therapeutic use - TU, toxicity - TO] |
| #11 | MeSH descriptor: [Sitagliptin Phosphate] explode all trees and with qualifier(s): [administration & dosage - AD, adverse effects - AE, metabolism - ME, pharmacokinetics - PK, pharmacology - PD, therapeutic use - TU, toxicity - TO] |
| #12 | MeSH descriptor: [Vildagliptin] explode all trees and with qualifier(s): [administration & dosage - AD, adverse effects - AE, metabolism - ME, pharmacokinetics - PK, pharmacology - PD, therapeutic use - TU, toxicity - TO] |
| #13 | MeSH descriptor: [Linagliptin] explode all trees and with qualifier(s): [administration & dosage - AD, adverse effects - AE, metabolism - ME, pharmacokinetics - PK, pharmacology - PD, therapeutic use - TU, toxicity - TO] |
| #14 | alogliptin |
| #15 | saxagliptin |
| #16 | dutogliptin |
| #17 | teneligliptin |
| #18 | gemigliptin |
| #19 | anagliptin |
| #20 | Trelagliptin |
| #21 | Retagliptin |
| #22 | Melogliptin |
| #23 | Evogliptin |
| #24 | Carmegliptin |
| #25 | LC15 0444 |
| #26 | #10 or #11 or #12 or #13 or #14 or #15 or #16 or #17 or #18 or #19 or #20 or #21 or #22 or #23 or #24 or #25 |
| #27 | #9 or #26 |
| #28 | MeSH descriptor: [Cognitive Dysfunction] explode all trees |
| #29 | MeSH descriptor: [Glucose Metabolism Disorders] explode all trees |
| #30 | MeSH descriptor: [Dementia] explode all trees |
| #31 | MeSH descriptor: [Alzheimer Disease] explode all trees |
| #32 | #28 or #29 or #30 or #31 |
| #33 | #9 and #32 |
| #34 | #26 and #32 |
| #35 | #27 and #32 |

**Web of science**

| **No.** | **Search strategies** |
| --- | --- |
| #46 | #43 AND #34 |
| #45 | #43 AND #33 |
| #44 | #43 AND #11 |
| #43 | #42 OR #41 OR #40 OR #39 OR #38 OR #37 OR #36 OR #35 |
| #42 | TS= 'vascular dementia' |
| #41 | TS='Mental Status' |
| #40 | TS=Dementia |
| #39 | TS='cognitive impairment' |
| #38 | TS='Cognition Disorders' |
| #37 | TS='Cognitive Dysfunction' |
| #36 | TS= 'cerebral glucose metabolism' |
| #35 | TS='Alzheimer Disease' |
| #34 | #33 OR #11 |
| #33 | #32 OR #31 OR #30 OR #29 OR #28 OR #27 OR #26 OR #25 OR #24 OR #23 OR #22 OR #21 OR #20 OR #19 OR #18 OR #17 OR #16 OR #15 OR #14 OR #13 OR #12 |
| #29 | TS=dutogliptin |
| #28 | TS=omarigliptin |
| #27 | TS=Carmegliptin |
| #26 | TS=evogliptin |
| #25 | TS=Melogliptin |
| #24 | TS=retagliptin |
| #23 | TS=Trelagliptin |
| #22 | TS=Diabegliptin |
| #21 | TS=teneligliptin |
| #20 | TS=anagliptin |
| #19 | TS=linagliptin |
| #18 | TS=alogliptin |
| #18 | TS=saxagliptin |
| #17 | TS=vildagliptin |
| #16 | TS=sitagliptin |
| #15 | TS= 'DPP4i' |
| #14 | TS='DPP IV inhibitOR' |
| #13 | TS='DPP 4 inhibitOR' |
| #12 | TS='Dipeptidyl-Peptidase IV Inhibitors' |
| #11 | TS='Dipeptidyl Peptidase 4 inhibitor' |
| #10 | #10 OR #9 OR #8 OR #7 OR #6 OR #5 OR #4 OR #3 OR #2 OR #1 |
| #9 | TS=semaglutide |
| #8 | TS=dulaglutide |
| #7 | TS=lixisenatide |
| #6 | TS=taspoglutide |
| #5 | TS=albiglutide |
| #4 | TS=liraglutide |
| #3 | TS=exenatide |
| #2 | TS='glucagon like peptide' |
| #1 | TS= 'Glucagon-Like Peptide-1 Receptor' |

**Psycinfo**

| **No.** | **Search strategies** |
| --- | --- |
| S3 | S1 OR S2 |
| S2 | "Dipeptidyl-Peptidase IV Inhibitors" OR "Dipeptidyl Peptidase 4 Inhibitors" "DPP 4 inhibitor" or "DPP IV inhibitor" OR dpp4 OR Sitagliptin OR Vildagliptin OR Linagliptin OR alogliptinOR saxagliptin OR dutogliptin OR teneligliptin OR gemigliptin OR anagliptin OR Trelagliptin OR Retagliptin OR Melogliptin OR Evogliptin OR Carmegliptin |
| S1 | "Glucagon-LikePeptide1" OR Exenatide OR Liraglutide OR albiglutide OR taspoglutide OR lixisenatide OR dulaglutide OR semaglutide |

**Embase**

| **No.** | **Search strategies** |
| --- | --- |
| #126 | #67 AND #119 |
| #125 | #66 AND #119 |
| #124 | #22 AND #119 |
| #123 | #109 AND #122 |
| #122 | #94 AND #119 |
| #121 | #89 AND #119 |
| #120 | #84 AND #119 |
| #119 | #110 OR #111 OR #112 OR #113 OR #114 OR #115 OR #116 OR #117 OR #118 |
|  |  |
| #118 | 'vascular dementia' |
| #117 | 'multiinfarct dementia' |
| #116 | 'dementia assessment' |
| #115 | 'cognitive impairment' |
| #114 | 'dementia' |
| #113 | 'cognitive defect' |
| #112 | 'cognitive defect' |
| #111 | 'cerebral glucose metabolism' |
| #110 | 'alzheimer disease' |
| #109 | #105 OR #106 OR #107 OR #108 |
| #108 | 'meta analysis (topic)' |
| #107 | 'systematic review (topic)' OR review |
| #106 | 'systematic review (topic)'/mj |
| #105 | 'meta analysis'/mj |
| #104 | #101 AND [embase]/lim |
| #103 | #100 AND [embase]/lim |
| #102 | #99 AND [embase]/lim |
| #101 | #98 AND ([article]/lim OR [article in press]/lim OR [conference abstract]/lim OR [conference paper]/lim) |
| #100 | #93 AND ([article]/lim OR [article in press]/lim OR [conference abstract]/lim OR [conference paper]/lim) |
| #99 | #88 AND ([article]/lim OR [article in press]/lim OR [conference abstract]/lim OR [conference paper]/lim) |
| #98 | #94 NOT #97 |
| #97 | #96 NOT #95 |
| #96 | #67 AND #83 AND [animals]/lim |
| #95 | #67 AND #83 AND [humans]/lim |
| #94 | #67 AND #83 |
| #93 | #89 NOT #92 |
| #92 | #91 NOT #90 |
| #91 | #66 AND #83 AND [animals]/lim |
| #90 | #66 AND #83 AND [humans]/lim |
| #89 | #66 AND #83 |
| #88 | #84 NOT #87 |
| #87 | #86 NOT #85 |
| #86 | #22 AND #83 AND [animals]/lim |
| #85 | #22 AND #83 AND [humans]/lim |
| #84 | #22 AND #83 |
| #83 | #68 OR #69 OR #70 OR #71 OR #72 OR #73 OR #74 OR #75 OR #76 OR #77 OR #78 OR #79 OR #80 OR #81 OR #82 |
| #82 | 'registration'/exp |
| #81 | 'registries'/exp |
| #80 | 'database'/exp |
| #79 | database* |
| #78 | 'control group'/exp |
| #77 | 'comparison group' |
| #76 | 'prospective studies'/exp |
| #75 | nonrandom |
| #74 | 'follow up studies'/exp |
| #73 | 'longitudinal studies'/exp |
| #72 | 'cohort studies'/exp |
| #71 | 'retrospective studies'/exp |
| #70 | 'case-control studies'/exp |
| #69 | random* |
| #68 | 'clinical trial'/exp OR 'controlled clinical trial'/exp OR 'randomized controlled trial'/exp |
| #67 | #1 OR #2 OR #3 OR #4 OR #5 OR #6 OR #7 OR #8 OR #9 OR #10 OR #11 OR #12 OR #13 OR #14 OR #15 OR #16 OR #17 OR #18 OR #19 OR #20 OR #21 OR #22 OR #23 OR #24 OR #25 OR #26 OR #27 OR #28 OR #29 OR #30 OR #31 OR #32 OR #33 OR #34 OR #35 OR #36 OR #37 OR #38 OR #39 OR #40 OR #41 OR #42 OR #43 OR #44 OR #45 OR #46 OR #47 OR #48 OR #49 OR #50 OR #51 OR #52 OR #53 OR #54 OR #55 OR #56 OR #57 OR #58 OR #59 OR #60 OR #61 OR #62 OR #63 OR #64 OR #65 OR #66 |
| #66 | #23 OR #24 OR #25 OR #26 OR #27 OR #28 OR #29 OR #30 OR #31 OR #32 OR #33 OR #34 OR #35 OR #36 OR #37 OR #38 OR #39 OR #40 OR #41 OR #42 OR #43 OR #44 OR #45 OR #46 OR #47 OR #48 OR #49 OR #50 OR #51 OR #52 OR #53 OR #54 OR #55 OR #56 OR #57 OR #58 OR #59 OR #60 OR #61 OR #62 OR #63 OR #64 OR #65 |
| #65 | 'gliptin'/exp |
| #64 | 'liptin' |
| #63 | 'januvia' |
| #62 | 'janumet' |
| #61 | 'juvisync' |
| #60 | 'galvus' |
| #59 | 'eucreas' |
| #58 | 'onglyza' |
| #57 | 'kombiglyze xr' |
| #56 | 'nesina' |
| #55 | 'oseni' |
| #54 | 'kazano' |
| #53 | 'trajenta' |
| #52 | 'gemiglo' OR 'qtern' OR 'vipidia' OR 'vipdoment' OR 'diabeglipt' OR 'elant' OR 'glucal' OR 'teneglucon' OR 'veriglip' OR 'glipten' OR 'suganon' |
| #51 | 'beskoa' |
| #50 | 'omarigliptin'/exp |
| #49 | 'da-1229' |
| #48 | 'tenelia' |
| #47 | 'lc15 0444' |
| #46 | 'carmegliptin'/exp |
| #45 | 'retagliptin' |
| #44 | 'evogliptin'/exp |
| #43 | 'melogliptin'/exp |
| #42 | 'pf-734200'/exp |
| #41 | 'trelagliptin'/exp |
| #40 | 'anagliptin'/exp |
| #39 | 'teneligliptin'/exp |
| #38 | 'dutogliptin'/exp |
| #37 | 'vildagliptin'/exp |
| #36 | 'saxagliptin'/exp |
| #35 | 'linagliptin'/exp |
| #34 | 'gemigliptin'/exp |
| #33 | 'sitagliptin'/exp |
| #32 | 'alogliptin'/exp |
| #31 | 'dppiv inhibitor*' |
| #30 | 'dpp4 inhibitor*' |
| #29 | 'dpp4 i' |
| #28 | 'dpp4i' |
| #27 | 'dpp iv inhibitor*' |
| #26 | 'dpp 4 inhibitor*' |
| #25 | 'dipeptidyl peptidase iv inhibitor'/exp |
| #24 | 'dipeptidyl peptidase iv inhibitors' |
| #23 | 'dipeptidyl peptidase iv inhibitor' |
| #22 | #1 OR #2 OR #3 OR #4 OR #5 OR #6 OR #7 OR #8 OR #9 OR #10 OR #11 OR #12 OR #13 OR #14 OR #15 OR #16 OR #17 OR #18 OR #19 OR #20 OR #21 |
| #21 | 'glp1 ra' |
| #20 | 'zp10a peptide 1' OR 'byetta' OR 'bydureon' OR 'victoza' OR 'lyxumia' OR 'adlyxin' OR 'tanzeum' OR 'eperzan' OR 'trulicity' OR 'ave 0010' OR 'ozempic' |
| #19 | 'zp10a peptide' |
| #18 | 'semaglutide'/exp |
| #17 | 'dulaglutide'/exp |
| #16 | 'lixisenatide'/exp |
| #15 | 'taspoglutide'/exp |
| #14 | 'albiglutide'/exp |
| #13 | 'liraglutide'/exp |
| #12 | 'exenatide'/exp |
| #11 | 'glp-1 ra*' |
| #10 | 'glp-1 agonists' |
| #9 | 'glp-1 agonist' |
| #8 | 'glucagon like peptide 1 receptor agonist' |
| #7 | 'glp-1 receptor agonist' |
| #6 | 'glucagon-like peptide-1 agonists' |
| #5 | 'glucagon receptor'/exp |
| #4 | 'glucagon like peptide 1 receptor agonists' |
| #3 | 'glp-1 receptor agonists' |
| #2 | 'glucagon like peptide 1'/exp |
| #1 | 'glucagon like peptide'/exp |

**Clinical trial**

| **No.** | **Search strategies** |
| --- | --- |
| #1 | exenatide OR liraglutide OR albiglutide OR taspoglutide OR lixisenatide OR dulaglutide OR semaglutide OR Byetta OR Bydureon OR Victoza OR Lyxumia OR Adlyxin OR Tanzeum OR Eperzan OR Trulicity OR ‘ZP10A peptide*’ OR ‘AVE 0010’ OR Ozempic |
| #2 | sitagliptin OR Januvia OR Janumet OR Juvisync OR vildagliptin OR Galvus OR Eucreas OR ‘Galvus Met’ OR saxagliptin OR Onglyza OR ‘Kombiglyze XR’ OR Qtern OR alogliptin OR Nesina OR Oseni OR Kazano OR Vipidia OR Vipdoment |
| #3 | linagliptin OR Trajenta OR gemigliptin OR Gemiglo OR anagliptin OR Beskoa OR teneligliptin OR Tenelia OR Diabeglipt OR Elant OR Glucal OR Teneglucon OR Veriglip OR Glipten OR Trelagliptin OR ‘PF 734200’ |
| #4 | retagliptin OR Melogliptin OR evogliptin OR Suganon OR Carmegliptin OR "LC15 0444" OR ‘DA-1229’ OR omarigliptin OR gliptin* OR dutogliptin |
| #1 and #2 and #3 and #4 |  |
