# Supplementary material for: Cognitive protection of incretin‐based therapies in patients with type 2 diabetes mellitus: A systematic review and meta‐analysis based on clinical studies
Source: J Diabetes Investig. 2023 May 5;14(7):864–73. doi: 10.1111/jdi.14015 (PMC10286783; doi:10.1111/jdi.14015)
Supplement: Supplementary file 4 — Table S2 | Cohort studies quality and risk of bias assessment (Newcastle Ottawa Quality Assessment Scale). [file JDI-14-864-s002.docx]

| Study ID | Representativeness of the exposed cohort | Selection of the non-exposed cohort | Ascertainment of exposure | Demonstration that outcome of interest was not present at start of study | Comparability of cohorts on the basis of the design or analysis | Assessment of outcome | Was follow-up long enough for outcomes to occur | Adequacy of follow up of cohorts | Total |
| --- | --- | --- | --- | --- | --- | --- | --- | --- | --- |
| Rizzo MR 2014 | * | * | * | - | * | * | * | * | 7 |
| Isik AT 2017 | * | * | * | * | * | * | * | - | 7 |
| Borzi AM 2019 | * | * | * | - | * | * | * | * | 7 |
| Bulut EA 2020 | * | * | * | - | * | * | * | * | 7 |

Supplementary Table 2 Cohort studies quality and risk of bias assessment (NOS)

^*^The scoring method of the table is: each item in the table gets a maximum of 1 point, if it meets the evaluation criteria, it gets 1 point, which is recorded as "*", otherwise, it does not score, which is recorded as "-". There are 8 evaluation items in total, with a full score of 8 points. NOS: Newcastle Ottawa Quality Assessment Scale
