# Supplementary material for: Cognitive protection of incretin‐based therapies in patients with type 2 diabetes mellitus: A systematic review and meta‐analysis based on clinical studies
Source: J Diabetes Investig. 2023 May 5;14(7):864–73. doi: 10.1111/jdi.14015 (PMC10286783; doi:10.1111/jdi.14015)
Supplement: Supplementary file 5 — Table S3 | Randomized controlled trials quality and risk of bias assessment (the Cochrane Collaboration's tool). [file JDI-14-864-s005.docx]

Supplementary Table 3 Randomized controlled trials quality and risk of bias assessment (the Cochrane Collaboration’s tool)

| Study ID | Random Sequence generation | Allocation concealment | Blinding of participants and personnel | Blinding of outcome assessment | Complete outcome data | Selective reporting | Other |
| --- | --- | --- | --- | --- | --- | --- | --- |
| Biessels GJ 2019 | low | unclear | double | low | low | low | high |
| Xue JJ 2020 | low | unclear | unclear | unclear | low | low | low |
| Biessels JL 2021 | low | low | double | low | low | low | high |
| Li Q 2021 | high | high | open-label | high | low | low | low |
